# Real-World Study of Tildrakizumab Survival in Psoriasis: Impact of Arthritis, Hypertension, and Prior Biologic Use

**DOI:** 10.3390/life15050789

**Published:** 2025-05-15

**Authors:** Raquel Santos-Juanes Galache, Sebastian Reyes García, Jimena Carrero Martín, Álvaro Nuñez Domínguez, Marta López Pando, Irene Álvarez Losada, Irene de la Fuente Villaverde, Ana Lozano-Blazquez, Esther Salgueiro, Javier Bordallo, Jorge Santos-Juanes, Cristina Galache Osuna

**Affiliations:** 1 Unidad de Gestion Clínica de Dermatología, Hospital Universitario Central de Asturias, 33011 Oviedo, Spain; raquel.santosjuanes@gmail.com (R.S.-J.G.); sebastian021314@gmail.com (S.R.G.); jimenacarrerom@gmail.com (J.C.M.); coke97nd@gmail.com (Á.N.D.); martalopezpando@gmail.com (M.L.P.); cristinagalache@gmail.com (C.G.O.); 2 Reumatology Department, Hospital Universitario de Santiago, 15706 Santiago de Compostela, Spain; irenealvarezlosada@gmail.com; 3 Unidad de Gestión Clínica de Farmacia, Hospital Universitario Central de Asturias, 33011 Oviedo, Spain; irenefv@mundo-r.com (I.d.l.F.V.); analozanob@icloud.com (A.L.-B.); 4 Área de Farmacología, Departmento de Medicina, Universidad de Oviedo, 33006 Oviedo, Spain; salgueiroesther@uniovi.es (E.S.); bordallojavier@uniovi.es (J.B.); 5 Área de Dermatología, Departamento de Medicina, Universidad de Oviedo, 33006 Oviedo, Spain; 6 GRIDER—Grupo de Investigación en Dermatología, Universidad de Oviedo, 33006 Oviedo, Spain; 7 Instituto de Investigación Sanitaria del Principado de Asturias (ISPA), 33011 Oviedo, Spain

**Keywords:** tildrakizumab, Anti-IL23, real world

## Abstract

In routine clinical settings, identifying the factors that influence the persistence of biologic therapies is crucial for tailoring psoriasis management to individual patient profiles. This study aimed to evaluate the real-world drug survival of tildrakizumab in patients diagnosed with plaque psoriasis at the Dermatology Department of HUCA and to explore the clinical predictors of treatment discontinuation. We conducted a retrospective, hospital-based analysis involving 100 patients treated with tildrakizumab (Ilumetri^®^) between 1 January 2021 and 30 April 2024. Kaplan–Meier estimates were used to construct survival curves, and multivariate analyses were performed using Cox proportional hazards regression models. Both crude and adjusted hazard ratios (HRs) were calculated to assess potential differences across patient subgroups. The multivariate analysis identified statistically significant associations between reduced drug survival and the presence of psoriatic arthritis (*p* = 0.02), previous biologic exposure (*p* = 0.02), and arterial hypertension (*p* = 0.012). Other comorbidities did not demonstrate significant effects. The most common reasons for treatment discontinuation were primary inefficacy and suboptimal response in patients with arthritis. Overall, tildrakizumab demonstrated robust survival outcomes in this patient population, though diminished persistence was observed in those with prior biologic use, comorbid arthritis, and hypertension.

## 1. Introduction

Psoriasis is a chronic, immune-mediated skin condition with a multifactorial etiology, involving complex interactions between genetic predisposition and environmental triggers that culminate in immune system dysregulation [1,2]. The disease is marked by abnormal keratinocyte hyperproliferation and the infiltration of immune cells into both the epidermis and dermis. Globally, the prevalence of psoriasis is estimated to be approximately 2–4%, with reported rates varying significantly across different regions, ranging from 0.91% in the United States to 11.4% in Western populations. Discrepancies in psoriasis prevalence, reporting quality, and registration across regions lead to varied global incidence rates [3]. A peak incidence typically occurs between the ages of 20–30 and 50–60 years [4].

Clinically, psoriasis presents with a wide range of phenotypic variability in terms of lesion morphology, distribution, severity, and progression. It can be classified into several distinct subtypes, including plaque, guttate, erythrodermic, generalized pustular psoriasis, and palmoplantar pustulosis. Of these, plaque psoriasis is the most prevalent form, accounting for up to 90% of all cases [5]. The classical clinical manifestations of psoriasis consist of the presence of red, infiltrated plaques, covered with coarse, silvery scaling. Predilection sites include the elbows and knees, scalp, and periumbilical and lumbar regions, although any anatomical site might be affected. The clinical course of psoriasis is marked by frequent relapses with fluctuating rates [6]. Based on lesion distribution and extent, disease severity is categorized as mild, moderate, or severe.

Although the precise pathogenic mechanisms remain incompletely understood, substantial evidence supports a central role for the IL-23/Th17 axis, TNF-alpha, and the chemokine CCL20 in driving psoriatic inflammation [7]. Psoriatic arthritis, a related inflammatory joint disorder, occurs in approximately 5% to 30% of individuals with psoriasis and is more commonly observed in those with extensive cutaneous disease. It affects both sexes equally and typically manifests following the appearance of skin symptoms [8].

Tildrakizumab (Ilumetri^®^) is a fully humanized IgG1κ monoclonal antibody that received FDA approval in 2018 for the treatment of moderate-to-severe plaque psoriasis [9]. The standard dosing regimen includes subcutaneous administration of 100 mg at weeks 0 and 4, followed by maintenance dosing every 12 weeks. In patients weighing over 90 kg or in those with a high disease burden, a 200 mg dose may be considered for enhanced clinical effectiveness [10]. Mechanistically, tildrakizumab binds selectively to the p19 subunit of interleukin-23 (IL-23), thereby inhibiting its interaction with the IL-23 receptor on Th17 cells. This blockade prevents downstream activation of the JAK-STAT signaling pathway—specifically the phosphorylation of JAK2, TYK2, and STAT3—and ultimately reduces the transcription of RORγt and the subsequent differentiation of Th17 cells. This suppression leads to decreased production of IL-17A and IL-17F, key cytokines in keratinocyte activation and proliferation [11,12].

Drug survival refers to the duration over which a treatment remains effective, safe, and acceptable to the patient [13]. This metric not only reflects therapeutic effectiveness and tolerability but also captures aspects of patient satisfaction and adherence. In some cases, drug survival is also interpreted based on defined periods of treatment interruption—such as the 24-week threshold for ustekinumab, which corresponds to two dosing intervals [13,14].

Evaluating drug survival in real-world settings provides insights into long-term treatment outcomes under routine clinical conditions, where dose adjustments, concomitant therapies, and changes in patient preference are common—unlike the fixed protocols of clinical trials. Moreover, external factors such as drug cost, the availability of new therapies, and evolving prescribing behaviors may also impact drug survival, thereby necessitating caution when comparing results across different biologic agents [15,16].

The aim of this study was to assess the drug survival of tildrakizumab and to identify clinical predictors of treatment discontinuation in patients with plaque psoriasis managed at the Dermatology Department of HUCA.

## 2. Materials and Methods

We conducted a retrospective, single-center study at the Dermatology Department of HUCA, including patients treated between 1 February 2021 and 30 April 2024. The study protocol received approval from the Ethics and Research Committee of the Principality of Asturias, Spain (Ref. 2023-468). A total of 100 patients who received tildrakizumab (Ilumetri^®^) (Almirall, Barcelona, Spain) at doses of 100 mg or 200 mg (for body weight >90 kg) for the treatment of plaque psoriasis were included. Only patients receiving the drug for dermatological indications were considered.

Electronic medical records were reviewed to collect the following baseline data: sex, age, weight, height, family history of psoriasis (defined as the presence of psoriasis in at least one first-degree relative), age at disease onset, previous biologic exposure (classified as naïve or non-naïve), and presence of psoriatic arthritis (confirmed by a rheumatologist). Comorbid conditions such as hypertension, diabetes mellitus (DM), and dyslipidemia were also documented. Patients were classified as having these comorbidities if they reported a prior diagnosis; if such a diagnosis appeared in their clinical records; or if they were taking antihypertensive, antidiabetic, or lipid-lowering agents. Additionally, patients with a blood pressure > 135/85 mmHg at consultation were categorized as hypertensive.

Relevant laboratory parameters were assessed to define dyslipidemia. Patients with triglyceride levels > 150 mg/dL, total cholesterol > 200 mg/dL, or LDL cholesterol > 160 mg/dL were considered to have dyslipidemia. Body mass index (BMI) was calculated as weight (kg) divided by height squared (m^2^), and obesity was defined as BMI ≥ 30, following the World Health Organization (WHO) criteria.

Descriptive statistics were reported as absolute frequencies and percentages. Drug survival, defined as the duration from treatment initiation to permanent discontinuation, was calculated retrospectively. Treatment response was classified as primary failure when there was no initial clinical improvement, defined as failure to achieve a PASI 75 response after 16 weeks of treatment. Secondary failure was defined as loss of PASI 75 response during follow-up after initially achieving it beyond week 16. Lack of control of joint activity included either no improvement in pre-existing joint symptoms or the development of new-onset arthritis.

## 3. Results

### 3.1. Patient Characteristics

The study cohort comprised 100 patients, all identified as of White ethnicity. The baseline characteristics are detailed in Table 1. Notably, there was a high prevalence of individuals with a positive family history of psoriasis and early disease onset, suggesting a potential genetic predisposition within the sample population.

### 3.2. Drug Survival

**Figure 1:**
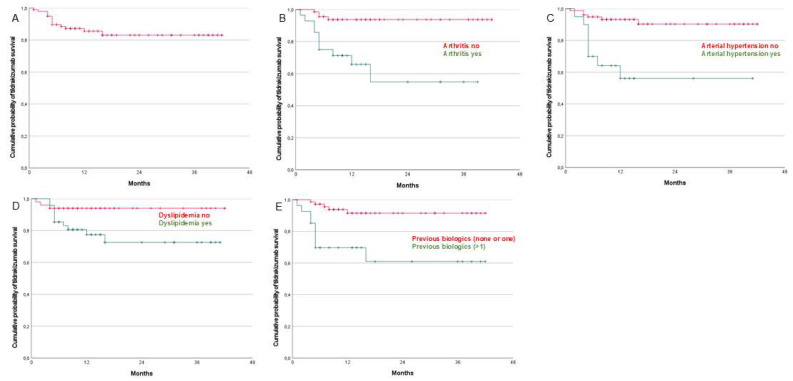
(**A**) Kaplan–Meier curve of tildrakizumab survival. (**B**) Kaplan–Meier curves of tildrakizumab survival according to arthritis (*p* < 0.001). (**C**) Kaplan–Meier curves of tildrakizumab survival according to arterial hypertension (*p* < 0.001). (**D**) Kaplan–Meier curves of tildrakizumab according to dyslipidemia (*p* < 0.028) (**E**) Kaplan–Meier curves of tildrakizumab survival according to previous biological treatments (*p* < 0.001).

The survival of the drug is shown in Figure 1.

In this cohort, the number of prior biologic therapies had a substantial impact on tildrakizumab survival at week 52. Drug persistence was 100% among biologic-naïve patients, declined to 93% in those with exposure to a single prior biologic, and further decreased to 67% in patients who had received two or more previous biologic agents (Table 2). These findings indicate a potential inverse association between the extent of prior biologic treatment and long-term persistence of tildrakizumab.

### 3.3. Univariate and Multivariate Analyses

Univariate analyses revealed statistically significant differences between subgroups based on the presence of psoriatic arthritis, hypertension arterial, and dyslipidemia and prior exposure to biologic therapies (Table 3). Patients with psoriatic arthritis had a 6.9-fold increased likelihood of treatment discontinuation compared to those without arthritis. Similarly, individuals with arterial hypertension exhibited a 6.3-fold higher risk of discontinuation relative to normotensive patients. Moreover, those with a history of more than one prior biologic therapy demonstrated a 5.6-fold greater risk of discontinuing tildrakizumab compared to patients who had received none or only one prior biologic agent.

In the univariate analysis according to the number of previously used biological treatments, the risk of discontinuation increases with each treatment line, with an unadjusted HR of 2.239, 95% CI (1.398–3.084)

### 3.4. Adverse Effects

All recorded adverse effects were mild: one patient with nasopharyngitis (1%), one patient with nausea after the first dose (1%), and one patient with an injection site reaction after the third dose (1%). No serious adverse effects were observed that required discontinuation of the drug.

### 3.5. Patients Discontinuing Treatment

At the end of the study, we observed that of the 100 patients who initiated treatment, 86 (86%) remained under follow-up. The 14 patients (14%) who discontinued treatment cited the following reasons: primary failure (8 patients), secondary failure (1 patient), and lack of control of joint activity (6 patients). One patient experienced both primary failure and an arthritis flare as reasons for discontinuation.

## 4. Discussion

This retrospective study evaluates the overall survival of tildrakizumab in a cohort of 100 patients treated at the Dermatology Department of HUCA. A notable strength of this investigation is the relatively large sample size drawn from a single tertiary care center, which enhances the consistency of clinical management and data collection. A recent study showed significant differences in survival rates among the different hospitals included in the study [17].

Real-world clinical practice studies of tildrakizumab have been published [18,19,20,21,22,23,24,25,26,27,28,29,30,31], but very few of the published ones report the survival of tildrakizumab in the treatment of psoriasis [17,32,33,34,35,36,37,38], and we did not find a single hospital series with this number of patients.

The demographic profile of our patient cohort is consistent with the findings reported in previous studies by Torres [31], Becher [32], Berenguer [17], and Melgosa [37], particularly regarding the predominance of male patients (54%) and the mean age at initiation of tildrakizumab therapy, which was 48 years.

The high proportion of bio-experienced patients is one of the most distinctive characteristics of our cohort (98.6%). In the literature, we found that studies on tildrakizumab were controversial and difficult to compare, given the different real-world evidence studies (97%), which showed much higher proportions than in studies such as that of Tsianakas (25%) [39] and o el de Narcisi (19%) [22]. These findings can be attributed to regional prescribing practices, as the local regulatory authority strongly advises that all patients with moderate-to-severe biologic-naïve psoriasis initiate treatment with a TNF inhibitor biosimilar as first-line therapy. In our series, there was no washout period between treatments [37]. Treatment with tildrakizumab was initiated immediately or within a few weeks after the discontinuation of the previous treatment.

The mean age of our patient cohort is similar to that of previous studies with TIL: 48.4 vs. 47.6 years [40], 47.8 years [39], and 51.4 years [34]. In our clinical practice, the mean age is also comparable to our series with other biologic drugs: ustekinumab (48 years) [41], adalimumab (46 years) [42], and secukinumab (50 years) [43].

Regarding the overall drug survival, the retention rates were 85% (first year), 81% (second year), and 81% (third year). The one-year drug survival observed in our study was at the upper end of the range reported in the literature, exceeded only by the findings of Ruiz-Villaverde, who reported a survival rate of 86% [35]. Our results were comparable to those of Torres [32] and notably higher than those reported by Becher (73%) [17] and Elgaard (64.3%) [36]. We observed that patients who reach one year tend to continue with the drug, as previously noted in the literature [17,44], especially if the patients do not have arthritis (89% in the first, second, and third years) or hypertension (90% in the first year and 85% in the second and third years).

Our analysis indicated that tildrakizumab survival was largely unaffected by the majority of patient- or disease-specific variables. Tildrakizumab survival was not affected by gender, obesity, age at treatment initiation, or disease duration, similar to findings reported in previous studies [22,32,35,45,46]. At this point, it is important to highlight the flexibility offered by the 200 mg dose of tildrakizumab as an effective therapeutic option, particularly for patients with high body weights, significant disease burden, or involvement of sensitive areas, as has been noted in previous studies [47,48]. We found statistically significant differences in the univariate analysis and in the Kaplan–Meier analysis regarding the use of prior biologics, and the presence or absence of arthritis, hypertension, and dyslipidemia. The latter lost its statistical significance in the multivariate analysis.

We found statistically significant differences between the use of a smaller number of previous biological lines of treatment (none or one) and a large number of previous biological lines of treatment from the Kaplan–Meier, univariate, and multivariate analyses, similar to what has been reported in the literature [17,20,30,32,35,49], though different from what other authors have noted [22,34,37].

We find it particularly interesting that, when introducing the variable for the number of previously used biologics, we observed in the univariate analysis that for each previously used biologic, the survival rate decreases by 22%, lower than what was found by Becher et al. [17]. Becher et al. stated that the number of previous biological treatments in the multivariate logistic regression model predicts that with every single additional treatment, the probability of the response to tildrakizumab decreases by 40% [17]. This is consistent with the findings of a greatest response when it was used as the first- or second-line biologic [17,20]. Conversely other studies have suggested that tildrakizumab response was not modified by prior biological therapy [30,37,50]. Our three naïve patients remain on treatment.

In this regard, we found a one-year survival rate similar to that reported by Ruiz-Villaverde and colleagues: naïve patients 100%, one prior biologic 92% vs. 90%, and more than one prior biologic 68% vs. 81% [35].

In our cohort, 28% of patients had psoriatic arthritis—a prevalence lower than that reported for other monoclonal antibody therapies used in our clinical setting, including ustekinumab (43%) [41], secukinumab (53%) [43], adalimumab (57%) [42], and apremilast (45%) [51]. This rate is comparable to those described by Ruiz (34%) [35] and Torres [32] but considerably higher than that reported by Melgosa (10%) [37]. In our series of biological treatments for psoriasis, only the survival of ustekinumab, an anti-IL12/23 molecule, decreases due to the presence of arthritis. In our series, 6 out of the 14 patients who discontinued treatment did so due to poor joint control (42.8%).

In the literature, we have not found a relationship between the presence of arthritis and differences in drug effectiveness, because the variable arthritis is not recorded, its relationship is not studied, or it is not statistically significant: Berenguer (17%) [34], Narcisi (15.2%) [22], Mastorino (16%) [49], Caldarola (16%) [23], Melgosa [37], Becher (21%) [17], and Di Brizzi (17.6%) [52]. At this point, it is important to note that our study has a high percentage of patients with arthritis diagnosed by a rheumatologist (28%), so the underrepresentation of this group in other studies could result in a type II error (the differences exist, but the low numbers prevent them from being detected). Alternatively, our patients may have more severe arthritis compared to those in other studies.

On the other hand, in our patients, the presence of hypertension predicts treatment discontinuation. We have not found this association in any other study. However, it has been reported that discontinuation rates are higher with an increasing number of comorbidities [17]. Conversely, in a 5-year study, no differences were found between patients with metabolic syndrome and those without it [53]. In a study evaluating risankizumab, an anti-IL-23 biologic, no significant differences in treatment outcomes were observed between patients with metabolic comorbidities and those without [54]. Interestingly, another study reported a better response in patients with a higher number of comorbidities, which was attributed to a worse initial PASI score in these patients [22].

Similar to what has been reported in the literature [55], we did not find differences in survival between patients younger and older than 65 years, in contrast to Chiricozzi et al., who found lower survival in patients over 65 years treated with anti-IL23 therapies [56].

Of the total number of patients, 14% discontinued treatment due to therapeutic failure, a lower rate than that reported by Becher (20%) [17]. The reasons were, primarily, primary failure (8%), followed by poor joint control (6%), and one patient with secondary failure (1%). While the observed rates of primary and secondary treatment failure are within the range of previously published results, there were no discontinuations due to serious adverse effects, similar to what has been described [22,57], and the number of discontinuations was lower than that in other studies, which range between 1% and 2% [17,34,40].

Regarding adverse effects, we found a rate of 3%, similar to those reported in the literature, which range from 5% [17,22] to 8.7% [40], and up to 15% in the study by Tsianakas [39]. Our findings align with pooled data from the reSURFACE clinical trials, real-world studies of tildrakizumab, and safety outcomes from other IL-23 inhibitor studies. These collectively report a low incidence of infections, minimal serious adverse events, and no significant signals for complications such as major cardiovascular events, malignancies, inflammatory bowel disease, or Candida infections [22,32].

## 5. Conclusions

Our data show that tildrakizumab survival in real-world clinical settings is in the higher range of published studies. The presence of arthritis, hypertension, and prior use of biologics reduces the drug’s survival. These findings, which have not been previously reported in the literature, need to be confirmed in prospective studies.

## Figures and Tables

**Table 1:** Baseline characteristics of patients.

Total Patients (n = 100)	
Sex (male), n (%)	54 (54%) male
Age at start of biologic treatment (years), mean ± SD	48.4 ± 15.6
Positive family history of psoriasis (yes), n (%)	63 (63%)
Onset before 40 years of age (%)	74 (74%)
Duration of treatment (months); mean ± SD	26.7 (IC95% = 23–29.72)
Initial PASI	13.2 ± 6.1
Tildrakizumab start age	48.23 ± 15.26
Age ≥ 65	16 (16%)
Comorbidities, n (%)	
Obesity (BMI ≥ 30)	40 (30%)
Diabetes mellitus	17%
Arterial hypertension	21 (21%)
Dyslipidemia	50 (50%)
Arthritis	28 (28%)
Prior treatments with biologics (%)	97 (97%)
One biologic	69 (69%)
Two biologics	18 (18%)
Three biologics	4 (4%)
Four biologics	3 (3%)
Five Biologics	3 (3%)

**Table 2:** Cumulative probability of tildrakizumab survival and cumulative probability according to arthritis status at different time intervals.

Percentage (95% CI)	1 Year	2 Years	3 Years	4 Years
Global	85 (78–93)	81 (71–91)	81 (71–91)	81 (71–91)
Arthritis no	93 (87–99)	93 (87–99)	93 (87–99)	93 (87–99)
Arthritis yes	68 (58–48)	54 (42–66)	54 (42–66)	54 (42–66)

**Table 3:** Cox regression analyses. Hazard ratios of risk of tildrakizumab discontinuation.

Univariate Analysis	HR (CI = 95%)	*p*-Value
Psoriasis onset ≥ 40 years 0.332	1.718 (0.575–5.135)	0.332
Sex (male) 0.810	1.137 (0.398–3.244)	0.810
Obesity: BMI ≥ 30	1.942 (0.674–5.598)	0.219
Arthritis: yes	6.851 (2.141–21,920)	0.001
Hypertension arterial: yes	6.275 (2.142–18.382)	<0.001
Dyslipidemia: yes	3.677 (1.026–13.185)	0.030
Family history: yes	1.134 (0.380–3.384)	0.822
Diabetes: yes	2.008 (0.628–6.413)	0.240
Previous biologics treatment > 1	5.581 (1.868–16.678)	<0.001
Older than 65	1.436 (0.400–5.153)	0.579
**Multivariate Analysis**	**HR (CI = 95%)**	***p*-Value**
Arthritis	4.098 (1.206–13.925)	0.024
Hypertension arterial	4.673 (1.410–15.488)	0.012
Previous biologic treatments > 1	3.704 (1.192–11.514)	0.024
Dyslipidemia	1.245 (0.299–5.178)	0.763

## Data Availability

The data presented in this study are available from the corresponding author on request.
